# The Herbal Medicine KBH-1 Inhibits Fat Accumulation in 3T3-L1 Adipocytes and Reduces High Fat Diet-Induced Obesity through Regulation of the AMPK Pathway

**DOI:** 10.1371/journal.pone.0142041

**Published:** 2015-12-09

**Authors:** Ji-Hye Lee, Taesoo Kim, Jung-Jin Lee, Kwang Jin Lee, Hyun-Kyu Kim, Bora Yun, Jongwook Jeon, Sang Kyum Kim, Jin Yeul Ma

**Affiliations:** 1 KM Application Center, Korea Institute of Oriental Medicine, Daejeon, 305–811, Republic of Korea; 2 College of Pharmacy, Chungnam National University, Daejeon, 305–764, Republic of Korea; 3 Nutraceutical Food R&D center, Kolmar BNH, 22–15 Sandan-gil, Jeonui-myeon, Sejong, 339–851, Republic of Korea; University of Bari Aldo Moro, ITALY

## Abstract

The aim of this study was to investigate whether a novel formulation of an herbal extract, KBH-1, has an inhibitory effect on obesity. To determine its anti-obesity effects and its underlying mechanism, we performed anti-obesity-related experiments *in vitro* and *in vivo*. 3T3-L1 preadipocytes were analyzed for lipid accumulation as well as the protein and gene expression of molecular targets involved in fatty acid synthesis. To determine whether KBH-1 oral administration results in a reduction in high-fat diet (HFD)-induced obesity, we examined five groups (n = 9) of C57BL/6 mice as follows: 10% kcal fat diet-fed mice (ND), 60% kcal fat diet-fed mice (HFD), HFD-fed mice treated with orlistat (tetrahydrolipstatin, marketed under the trade name Xenical), HFD-fed mice treated with 150 mg/kg KBH-1 (KBH-1 150) and HFD-fed mice treated with 300 mg/kg KBH-1 (KBH-1 300). During adipogenesis of 3T3-L1 cells *in vitro*, KBH-1 significantly reduced lipid accumulation and down-regulated the expression of master adipogenic transcription factors, including CCAAT/enhancer binding protein (C/EBP) β, C/EBP α and peroxisome proliferation-activity receptor (PPAR) γ, which led to the suppression of the expression of several adipocyte-specific genes and proteins. KBH-1 also markedly phosphorylated the adenosine monophosphate-activated protein kinase (AMPK) and acetyl-CoA carboxylase (ACC). In addition, KBH-1-induced the inhibition effect on lipid accumulation and AMPK-mediated signal activation were decreased by blocking AMPK phosphorylation using AMPK siRNA. Furthermore, daily oral administration of KBH-1 resulted in dose-dependent decreases in body weight, fat pad mass and fat tissue size without systemic toxicity. These results suggest that KBH-1 inhibits lipid accumulation by down-regulating the major transcription factors of the adipogenesis pathway by regulating the AMPK pathway in 3T3-L1 adipocytes and in mice with HFD-induced obesity. These results implicate KBH-1, a safe herbal extract, as a potential anti-obesity therapeutic agent.

## Introduction

Obesity, a disorder that affects the balance between energy intake and expenditure, is characterized by an excessive accumulation of body fat in white adipose tissue and marked adipocyte dysfunction [1, 2]. Adenosine-monophosphate-activated protein kinase (AMPK) is involved in adipose tissue, liver, muscle, heart and brain in the maintenance of cellular as well as body energy homeostasis [3, 4]. The phosphorylated form of AMPK inhibits metabolic enzymes involved in fatty acid synthesis, such as acetyl-CoA carboxylase (ACC), and regulation of adipogenesis [5–7].

Adipogenesis from preadipocytes into mature adipocytes is a complex process coordinated by the integration of many different signaling pathways and transcription factors. In particular, the nuclear hormone receptor peroxisome proliferator-activated receptor gamma (PPAR γ) and CCAAT-enhancer-binding protein (C/EBP) families are known to be major adipogenic transcription factors. During the initial stage of adipocyte differentiation, C/EBP β and C/EBP δ are rapidly induced; these cells then undergo mitotic clonal expansion followed by transcriptional activation of C/EBP α and PPAR γ, two major late acting adipogenic transcription factors. Induction of C/EBP α and PPAR γ results in the expression of many genes involved in the differentiation and maturation of these cells into adipocytes. These genes include those encoding adipokines (adiponectin and leptin), lipid metabolizing enzymes (fatty acid binding protein (Fabp) 4), lipoprotein lipase (LPL), fatty acid synthase (FAS) and insulin receptor (IR), all of which are up-regulated by these transcription factors during adipocyte differentiation [8–10].

Extensive research efforts have been devoted to developing pharmaceutical anti-obesity drugs. Although various medications have been used to treat obesity in patients, there is a limit to their long-term application because of severe side effects [11]. Only orlistat (tetrahydrolipstatin, marketed under the trade name Xenical) has been approved by the FDA for long-term use. Recently, many studies have demonstrated anti-obesity activity for natural products and suggested their potential as anti-obesity agents or supplements [12, 13]. A mixed herb formulation may be able to deliver synergistic therapeutic effects, leading to maximal therapeutic efficacy with minimal adverse effects [14–16]. We developed a novel mixed herbal medicine, KBH-1, as a potential safe and effective anti-obesity drug. KBH-1 consists of *Polygala tenuifolia*, *Curcuma longa* and *Saururus chinensis*. Each herb has been used in traditional Korean medicine and in other countries for a variety of medicinal purposes [17–24]. However, the direct and synergistic effects of *Polygala tenuifolia*, *Curcuma longa*, and *Saururus chinensis* extracts on obesity and adipogenesis have not yet been examined. In this study, the anti-obesity effects of KBH-1 and its molecular mechanism of action were investigated in 3T3-L1 mouse adipocytes and in high fat diet (HFD)-induced obese mice. Our results provide scientific support for the medical use of a new mixed herbal extract in treating obesity.

## Materials and Methods

### Materials and Reagents

*Polygala tenuifolia* Willdenow, *Curcuma longa* Linne and *Saururus chinensis* Baill were obtained from Yeongcheon Oriental Herbal Market (Yeongcheon, Korea) (Table 1). 3T3-L1 cells were obtained from the American Type Culture Collection (Manassas, VA). 3-isobutyl-1-methylxanthine (IBMS), dexamethasone and insulin were purchased from Sigma Chemical (St. Louis, MO, USA). Penicillin, bovine serum, streptomycin and fetal bovine serum were purchased from Gibco (Carlsbad, CA, USA). AdipoRed and Dulbecco’s modified Eagle’s medium (DMEM) were obtained from Lonza (Walkersville, MD, USA). Primary antibodies for immunoblot analysis were purchased from Cell Signaling Technology, Inc. (Boston, MA, USA). Secondary antibodies for immunoblot analysis, the ECL kit and the BCA protein assay kit were purchased from Thermo (Rockford, IL, USA). The PVDF membrane was purchased from Millipore (Darmstadt, Germany). The RNA extraction kit was purchased from Ambion (Austin, Texas, USA). The AccuPower GreenStar qPCR Master Mix, the AccuPower RT PreMix and primers were supplied from Bioneer (Daejeon, Korea). Male C57BL/6N mice were obtained from Samtako (Osan, Korea) and the 10% and 60% kcal fat diets were purchased from Research Diet, Inc. (New Brunswick, USA). The leptin ELISA kit was purchased from Mediagnost (mouse and rat leptin ELISA kit; Germany) and the ghrelin ELISA kit was purchased from SCETI (active/desacyl-ghrelin ELISA kit; Japan).

**Table 1:** Herbal composition of KBH-1.

Herbal product	Scientific name	Amount (g)	Location of origin
Saururi Herba	*Saururus chinensis* (Lour.) Baill.	600	Yeongcheon, Korea
Polygala Root	*Polygala tenuifolia* WILLD.	600	Yeongcheon, Korea
Curcuma Longa Rhizome	*Curcuma longa* L.	600	Yeongcheon, Korea

### KBH-1 Preparation

KBH-1 consists of *Polygala tenuifolia*, *Curcuma longa* and *Saururus chinensis* at a ratio of 1: 1: 1. All voucher specimens were stored in the herbal bank of the KM-Based Herbal Drug Research Group, Korea Institute of Oriental Medicine. Each herb was added to 60% ethanol (18 and 9 kg) and then extracted by heating at 70–75°C twice (3 h and 2 h) by a COSMOS-660 (Kyungseo Machine Co, Korea). After extraction, KBH-1 extract was filtered using a 50-μm nylon mesh and concentrated using Laborota 20 (Heidolph Ins., Germany). The concentrate was made into a powder by freeze-drying. The yield rate was 20.25 ± 2.5%.

### Cell Culture and Differentiation

Cell culture and differentiation of adipocytes was performed as described previously [25]. Briefly, for differentiation, 3T3-L1 cells were cultured to full confluence in growth medium (GM) consisting of DMEM supplemented with 10% bovine serum and penicillin (100 U/ml)-streptomycin (10 μg/ml) at 37°C in a humidified atmosphere with 5% CO_2_ in an incubator. After reaching approximately 90% confluence (referred to as day 0), the cells were switched over to differentiation medium (DM) consisting of DMEM with 10% fetal bovine serum, insulin (1 μg/ml), dexamethasone (1 μM) and IBMX (0.5 mM), and then cultured for 3 days. Next, the cells were maintained in DM containing only insulin (10 μg/ml) and the medium was changed daily for 2 days. In this culture condition, mature adipocytes normally differentiated after day 7. To investigate the effects of KBH-1 on adipocyte differentiation, KBH-1 was administered until the cells were harvested, and then the intracellular triglyceride content was measured using Adipored according to the manufacturer’s protocol. Cytotoxicity was tested using the WST-1 kit (CCK-8; Dojindo Molecular Technologies, Rockville, MD, USA) according to the manufacturer’s protocol. In addition, to identify the effect of KBH-1 on adipocyte accumulation, fat droplets in 3T3-L1 cells were stained with Oil red O dye and were then examined using a Nikon digital camera system.

### siRNA Transfection

The AMPK-specific siRNA (si-AMPKα1/2) oligonucleotide was purchased from Santa Cruz biotechnology, Inc. (CA, USA). si-AMPK was transfected into cells using TranslT-X2 (Mirus Bio LLC, Madison, USA) according to the manufacture’s protocol. Briefly, final concentration 50 nM si-AMPK were incubated with 3T3-L1 preadipocyte for 72 h, and then transfection medium was removed and cells were differentiated in the same condition as normal differentiation.

### Preadipocyte Proliferation and Cell Viability

An MTT (3-(4,5-dimethylthiazol-2-yl)-2,5-diphenyltetrazolium bromide) assay was performed as described previously [26]. Briefly, 3T3-L1 preadipocytes were seeded at a 1× 10^3^ cells/well in 96-well plates. The cells were cultured in GM with KBH-1 of various concentrations for 24 and 72 hours. MTT solution (2.5 mg/ml) was added to the medium, and cells were incubated for 3 hours. The purple formazan crystals were dissolved in DMSO (dimethyl sulfoxide), and the absorbance was read at 570 nm on the infinite M200 plate reader (TECAN Group Ltd., Männedorf, Switzerland).

### Flow Cytometry Analysis

3T3-L1 preadipocytes were differentiated in the presence of KBH-1 at a concentration of 10 μg/ml for 24 hours. The cells were harvested and fixed overnight with 80% ethanol at 4°C and then washed with PBS containing EDTA. Next, the cells were stained with propidium iodide (PI) solution containing RNase for 30 min. The PI-stained DNA complex was measured with a FACsCalibur (Becton-Dickinson Co., Franklin Lakes, NJ, USA).

### Real-Time Polymerase Chain Reaction (RT-PCR)

RT-PCR analysis was performed as described previously [25, 27]. Total cellular RNA was extracted using TRIzol reagent according to the instructions provided by the supplier. Isolated RNA was assessed using a nano-drop spectrophotometer (Thermo Scientific, Ltd., Waltham, MA) and cDNA synthesis was performed using the AccuPower RT PreMix cDNA synthesis kit according to the manufacturer’s protocol. Subsequently, SYBR green-based qPCR amplification was performed using cDNA, 10 pM of primers and the AccuPower GreenStar qPCR Master Mix in the Applied Eco Real-time PCR system (Illumina, Inc., San Diego, CA, USA) according to the manufacturer’s protocol. The primer sequences for PCR analysis were as follows: CD36 (sense) GCTTGCAACTGTCAGCACAT, (antisense) GCCTTGCTGTAGCCAAGAAC; leptin (sense) CCACACACAGCTGGAAACTC, (antisense) GCCTTGCTTCAGATCCATCC; PPAR γ (sense) TGATGGAAGACCACTCGCAT (antisense) CCATCCTTCAC-AAGCATGAA; C/EBP β (sense) GTTTCGGGAGTTGATGCAATC, (antisense) AACAACCCCGCAGGAACAT; C/EBP α (sense) GTGTGCACGTCTA-TGCTAAACCA, (antisense) GCCGTTAGTGAAGAGTCTCAGTTTG; FAS (sense) TGGTGGGTTTGGTGAATTGTC, (antisense) GCTTGTCCTGCTCTAACTGGAAGT; Fabp4 (sense) CCAATGAGCAAGTGGCAAGA, (antisense) GATGCC-AGGCTCCAGGATAG; LPL (sense) GGCCAGATTCATCAACTGGAT, (antisense) GCTCCAAGGCTGTACCCTAAG; adiponectin (sense) GGAGATGCAGGTCTTCTTGGT, (antisense) TCCTGA- TACTGGTCGTAGGTGAA; 18srRNA (sense) CATTCGAACGTCTGCCCTATC, (antisense) CCTGCTGCCTTCCTTGGA; and β-actin (sense) TGTCCACCTTCCAGCAGATGT, (antisense) AGCTCAGTAACAGTCCGCCTAGA. The PCR reaction consisted of three segments [28]. In the first segment, the polymerase was activated by heating at 95°C for 10 min. The second segment consisted of 40 cycles at 94°C for 10 sec, 60°C for 30 sec and 72°C for 30 sec. The third segment consisted of 95°C for 15 sec, 55°C for 15 sec and 95°C for 15 sec. All reactions were run in triplicate and data were analyzed using the 2^-(ave.∆∆CT)^ method.

### Protein Extraction and Immunoblotting

Immunoblotting was performed as described previously [28]. The harvested cells were lysed in RIPA buffer containing 50 mM tris-HCl (pH 8.0), 5 mM EDTA, 150 mM NaCl, 1% NP-40, 0.1% SDS, 1 mM PMSF, protease-inhibitor cocktail tablet and phosphatase-inhibitor cocktail tablet. Cell lysates were centrifuged at 13,000 rpm for 30 min at 4°C. Protein concentration was determined with a BCA Protein Assay Kit. Protein samples were mixed with sample buffer (100 mM Tris-HCl [pH 7.6], 2% SDS, 1% 2-mercaptoethanol, 2% glycerol and 0.01% bromophenol blue) and incubated at 97°C for 5 min. Protein extracts in an amount of 15 μg per sample were loaded onto 8~15% polyacrylamide gels. Electrophoresis was performed using the Mini Protein 3 Cell (Bio-Rad, Hercules, CA, USA). Resolved proteins were transferred onto a PVDF membrane. The membrane was incubated in blocking buffer (3% BSA with 10 mM Tris-HCl, 150 mM NaCl, and 0.1% Tween 20) and then incubated overnight at 4°C with diluted (1: 1000) primary antibodies. After washing with buffer (10 mM Tris-HCl, 150 mM NaCl and 0.1% Tween 20) three times for 20 min each time, the membrane was probed with diluted (1: 5000) secondary antibodies for 1 h at room temperature. The membrane was then washed three times for 10 min each time and developed with an ECL kit. Chemiluminescent signals were detected using a LAS-4000 Luminescent Image Analyzer (Fuji Photo Film Co., Japan). Band intensities were normalized to β-actin or the respective total proteins and quantified using ImageJ software (National Institutes of Health, USA).

### High-Fat Diet (HFD)-Induced Obesity Mouse Model

All animal experiments were performed according to the Guide for the Care and Use of Laboratory Animals of the National Institutes of Health (NIH publication No. 83–23, revised 1996) and were approved by the Institutional Animal Care and Use Committee of KIOM. Male C57BL/6N mice were purchased from Samtako (Osan, Korea). Mice were housed in a room with controlled temperature (20–24°C), humidity (40–60%) and lighting (12 h light/dark cycle) and were supplied with water *ad libitum*. After acclimation for 1 week, mice were randomly divided into five groups of nine mice each: (1) ND: 10% kcal fat diet fed mice; (2) HFD: 60% kcal fat diet fed mice; (3) orlistat: HFD treated with 75 mg/kg/day orlistat administration; (4) KBH-1 150: HFD treated with 150 mg/kg/day KBH-1 extract administration; and (5) KBH-1 300: HFD treated with 300 mg/kg/day KBH-1 extract administration. Mice were provided with 10% kcal fat or 60% kcal fat diets for 8 weeks. Drugs or herbal extracts were administered orally once a day during the diet period. The same amount of saline was orally administered to the control groups. The animal monitoring to assess animal body condition and health was performed once a day. Also, body weight of animals was weighed once a week. At the end of the experimental period, mice were euthanized by intraperitoneal injection of Avertin (500 mg/kg, tribomoethanol), blood was collected, and organs were excised. The organs were rinsed with saline solution, weighed and stored at -80°C.

### High Performance Liquid Chromatography (HPLC) Analysis of KBH-1 and Its Standard Compounds

KBH-1 was standardized for quality control by HPLC analysis using standard reference compounds (rutin, quercitrin, onji-saponin B, bisdemethoxycurcumin (BDMC), demethoxy-curcumin (DMC) and curcumin). Chromatographic analysis of KBH-1 and standard compounds was performed on a reverse-phase HPLC system (Dionex Co., CA, USA) equipped with an ultimate 3000 pump, ultimate diode array detector (DAD), injector 10 μL sample loop (Dionex, ID × L 0.18 × 550 mm Viper 550 mm USA) and Chromeleon data acquisition system (Dionex, version 7). Separation was performed using an Optimapak C_18_ RP-column (250×4.6 mm, 5 μm, C_18_, Korea) at 40°C. A gradient elution was performed using the following solvent systems: mobile phase A: double distilled water/triflouroacetic acid (TFA) (99.9/0.1; v/v %), mobile phase B: acetonitrile. The run time was 70 min, and the linear gradient method was applied to the mobile phase condition (solvent A: 0–70 min; 90–10, B: 30–70%). The elution was performed with a gradient procedure as follows: 0–7 min, 2% B; 2–60 min, from 2% B to 98% B.

### Statistical Analysis

Values are expressed as the mean ± standard error (SEM). The statistical significance between each group was analyzed using one-way ANOVA. Differences were analyzed using the Bonferroni test. All analyses were performed using SPSS ver. 21 (SPSS Inc., Chicago, IL, USA).

## Results

### KBH-1 Inhibits Lipid Accumulation in 3T3-L1 Adipocytes

**Fig 1:**
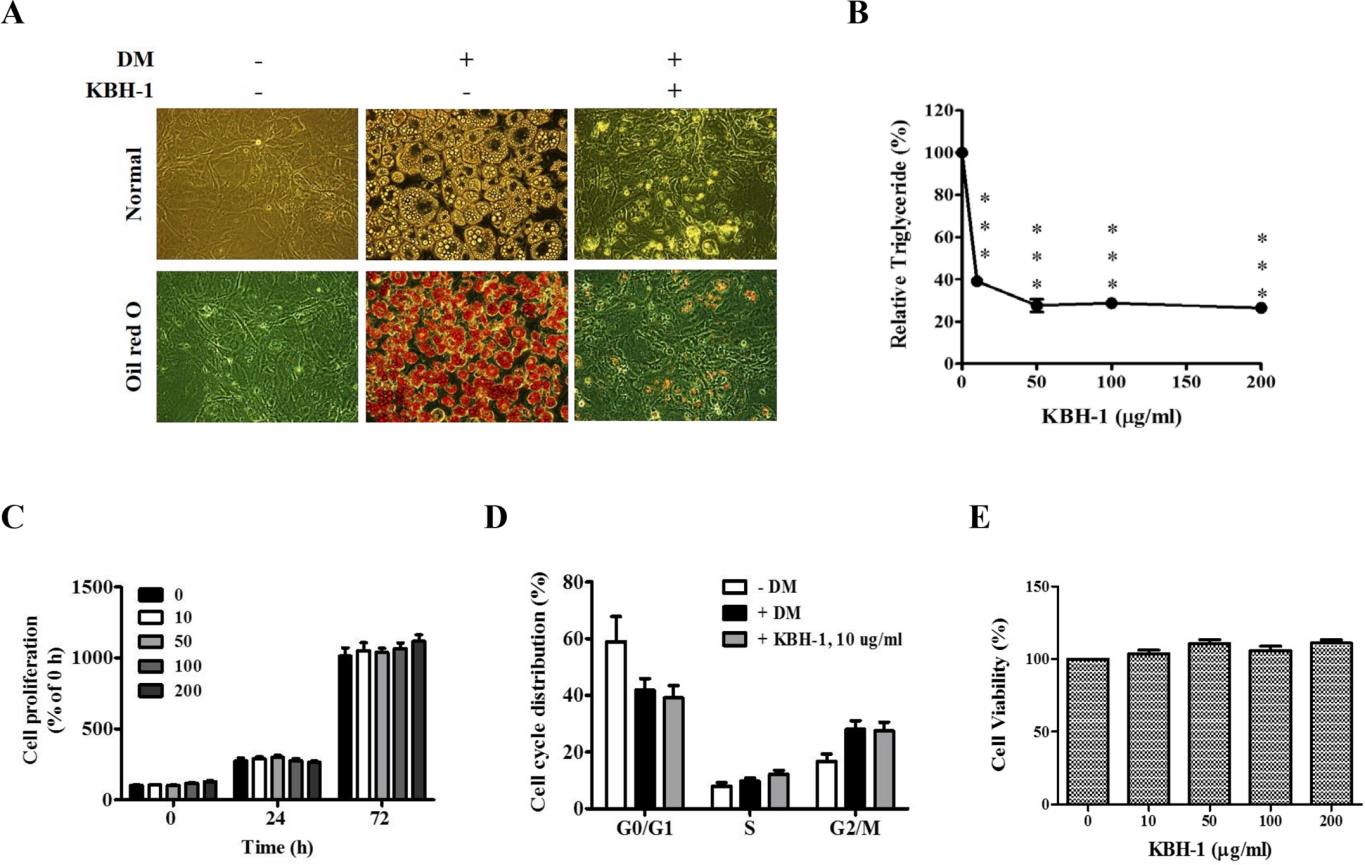
Effects of KBH-1 on lipid accumulation and proliferation in 3T3-L1 adipocyte. 3T3-L1 preadipocytes were induced to differentiate into mature adipocytes in the presence of KBH-1. **(A)** Lipid accumulation was measured using Oil Red O staining at a concentration of 10 μg/ml KBH-1, **(B)** intracellular triglyceride (TG) content was measured using the AdipoRed assay in the presence of KBH-1 (0–200 μg/ml). **(C)** Cell proliferation was examined using MTT assay for 24 and 72 h in the presence of KBH-1 (0–200 μg/ml). **(D)**Cell cycle distribution was evaluated or 24 h in the presence of 10 μg/ml of KBH-1 using flow cytometry. **(E)** Cell viability was examined on day 7 using cell count kit-8. Data are expressed as the mean ± SEM. Significant differences from control (0 μg/ml) are indicated by ***p < 0.001.

To examine the effect of KBH-1 on adipocyte differentiation, 3T3-L1 preadipocytes were cultured in differentiation medium (DM) for 7 days, which induced their differentiation into mature adipocytes and resulted in significant accumulation of intracellular lipid droplets. These lipid droplets were stained with Oil red O, and the intracellular triglycerides were quantified. As shown in Fig 1A, treatment with 10 μg/ml KBH-1 markedly reduced intracellular lipid droplet accumulation. Furthermore, the triglyceride content was also significantly decreased by treatment with KBH-1 in a dose-dependent manner (Fig 1B).

### Effect of KBH-1 on Preadipocyte Proliferation, Cell Cycle and Viability

To investigate the effect of KBH-1 on preadipocyte proliferation, 3T3-L1 preadipocytes were grown in GM medium supplemented with different concentrations of KBH-1 for 0, 24 and 72 hours, respectively. As shown in Fig 1C, KBH-1 treatment did not affect cell preadipocyte proliferation up to a concentration of 200 μg/ml. Differentiating 3T3-L1 cells treated with a 10 μg/ml dose of KBH-1 were subjected to flow cytometry. As shown in Fig 1D, mitotic clonal expansion in 3T3-L1 preadipocytes was induced by MDI, but KBH-1 did not affect the cell cycle.

To exclude the possibility that the inhibitory effect of KBH-1 on adipocyte differentiation was due to cytotoxicity, a cell viability test was conducted after treatment with various concentrations of KBH-1 for 7 days. As shown in Fig 1E, KBH-1 did not affect cell viability up to a concentration of 200 μg/ml. This result suggests that the inhibitory effects of KBH-1 on fat droplet formation and triglyceride accumulation were not due to cytotoxicity.

### KBH-1 Decreases Protein and Gene Expression of Adipogenic Transcription Factors

**Fig 2:**
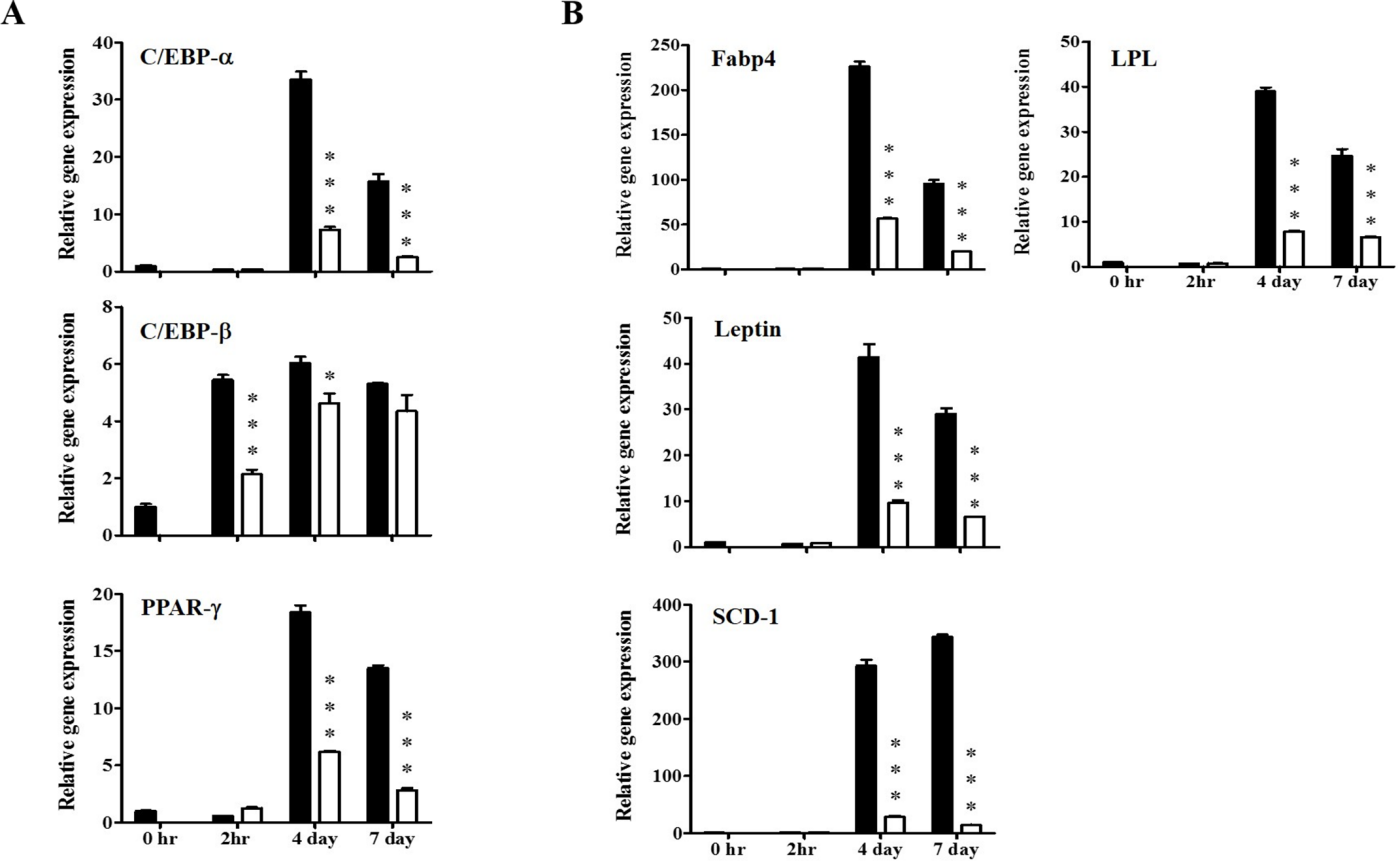
Effects of KBH-1 on the gene expression of adipogenic-related factors and specific markers. 3T3-L1 preadipocytes differentiated for 30 min, 1 and 2h, or 2 h, 4 and 7 days in the absence or presence of 10 μg/ml KBH-1. Gene expression of **(A)** adipogenic-related factors, such as C/EBP α, C/EBP β, and PPAR γ, and **(B)** adipogenic-specific factors, such as fatty acid binding protein 4 (Fabp4), lipoprotein lipase (LPL), SCD-1 and leptin, were analyzed by quantitative real-time PCR. Results are expressed relative to untreated cells after normalization to β-actin mRNA levels. Data are expressed as the mean ± SEM. Significant differences from each time-point control (no KBH-1 treatment) are indicated by *p < 0.05 or ***p < 0.001. ■; 0 μg/ml, KBH-1 □; 10 μg/ml KBH-1.

**Fig 3:**
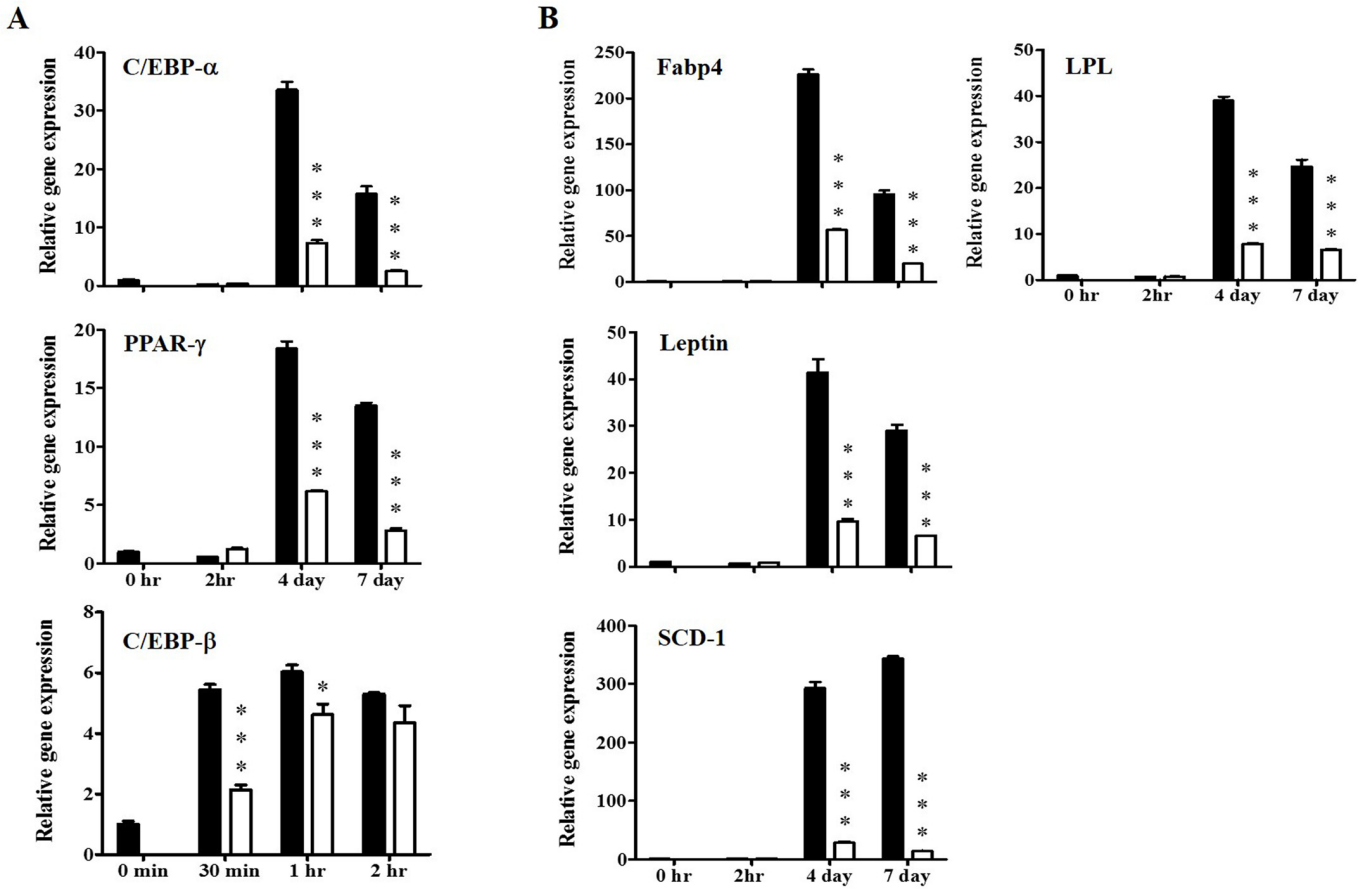
Effects of KBH-1 on the activation of adipocyte-specific proteins in 3T3-L1 cells differentiation. 3T3-L1 preadipocytes differentiated for 2 h, 4 and 7 days in the absence or presence of 10 μg/ml KBH-1. Adiponectin, leptin, fatty acid synthase (FAS), fatty acid binding protein 4 (Fabp 4), lipoprotein lipase (LPL) and insulin receptor (IR) were determined by Western blotting. Results are expressed relative to untreated cells after normalization to β-actin protein levels. Data are expressed as the mean ± SEM. Significant differences from each time-point control (no KBH-1 treatment) are indicated by ***p < 0.001. ■; 0 μg/ml KBH-1, □; 10 μg/ml KBH-1.

To identify the effect of KBH-1 on adipogenic transcription factors and adipocyte-specific gene expression, 3T3-L1 cells were cultured with adipocyte differentiation medium (DM) in the presence or absence of KBH-1 (10 μg/ml). Studies of adipogenic cell lines have shown that C/EBP β, PPAR γ and C/EBP α, as key adipogenic transcription factors, are induced during adipocyte differentiation. The mRNA levels of C/EBP β, induced by DM, increased within 30 min, reached a maximum at 1 h, and then decreased, whereas KBH-1 significantly inhibited C/EBP β mRNA expression at 30 min and at 1 h. The mRNA levels of C/EBP α and PPAR γ were also significantly decreased compared to the control after 4 and 7 days (Fig 2A). KBH-1 treatment also led to the suppression of PPAR γ target gene expression, including Fabp4, LPL, SCD-1 and leptin (Fig 2B). Adipogenic markers (FAS, adiponectin, leptin, LPL and Fabp4) were also measured during differentiation of 3T3-L1 cells. As shown in Fig 3, KBH-1 markedly suppressed the protein levels of FAS, Fabp4, LPL, adiponectin, leptin and IR (insulin receptor) at 4 and 7 days.

### KBH-1 Regulates the AMPK Pathways in 3T3-L1 Cells

**Fig 4:**
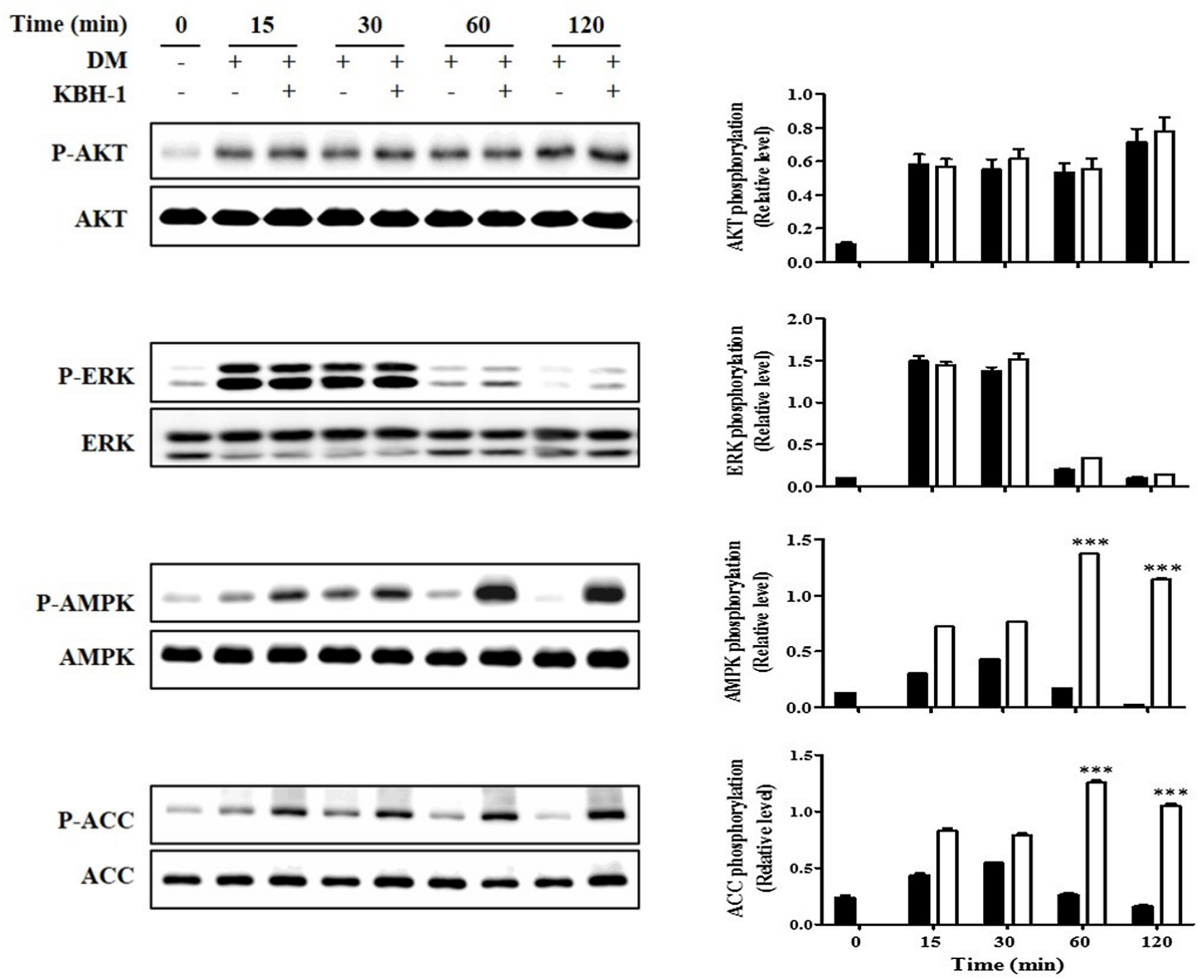
Effects of KBH-1 on AKT, ERK1/2, AMPK and ACC phosphorylation. 3T3-L1 preadipocytes differentiated for 15, 30, 60 and 120 min in the absence or presence of 10 μg/ml KBH-1. AKT, ERK1/2, AMPK and ACC phosphorylation were measured using SDS-PAGE and immunoblotting. Bar graph (right panel) is the relative density after normalization to total form of each protein. Data are expressed as the mean ± SEM. Significant differences from each time-point control (no KBH-1 treatment) are indicated by *** *p* < 0.001. ■; 0 μg/ml KBH-1, □; 10 μg/ml KBH-1.

**Fig 5:**
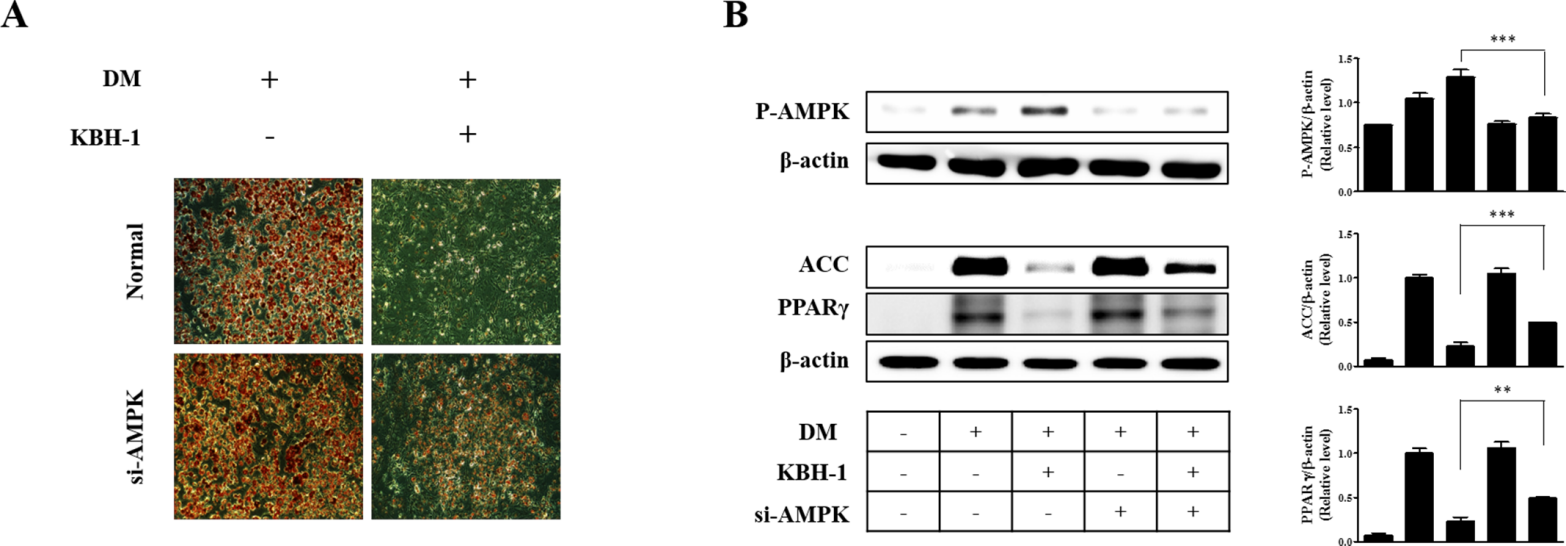
Effects of siRNA for AMPK on KBH-1-induced inhibition of adipocyte differentiation. 3T3-L1 preadipocytes were induced to differentiate into mature adipocytes in the presence of KBH-1. Final concentration of 50 nM si RNA for AMPK (si-AMPK) was incubated with 3T3-L1 preadipocyte for 72 h, and then transfection medium was removed and cells were differentiated in the same condition as normal differentiation. **(A)** Lipid accumulation was measured using Oil Red O staining at a concentration of 10 μg/ml KBH-1 on day 7. (B) AMPK, ACC and PPARγ phosphorylation were measured using SDS-PAGE and immunoblotting. Bar graph (right panel) is the relative density after normalization to β-actin. Data are expressed as the mean ± SEM. Significant differences from the band of no si-AMPK treatment in the presence of KBH-1 are indicated by ** *p* < 0.01.

In the insulin signaling pathway, AKT, ERK1/2 and AMPK are upstream of adipocyte differentiation pathways including the PPAR γ and C/EBP α pathways [25]. Therefore, in this study, to evaluate the effect of KBH-1 on signaling pathways upstream of PPAR γ and C/EBP α, we investigated the effects of KBH-1 on the levels of phosphorylated AKT, ERK1/2 and AMPK. In 3T3-L1 preadipocytes, AKT and ERK1/2 were both phosphorylated to a significant extent during the early stage of adipogenesis, but treatment with KBH-1 did not affect phosphorylation of ERK1/2 and AKT. Treatment with KBH-1 significantly increased phosphorylation of AMPK compared with the control during the early stage of adipogenesis. Phosphorylation of AMPK led to the inactivation of lipogenic enzymes such as ACC. Treatment with KBH-1 significantly phosphorylated the ACC compared with the control (Fig 4). These results indicate that phosphorylated AMPK lead to the inactive form of ACC, since ACC become inactive after phosphorylation. In addition, to identify the synergic effect of KBH-1 through AMPK activation, KBH-1 or an extract from *Polygala tenuifolia*, *Saururus chinensis* or *Curcuma longa*, was added to adipocyte differentiation medium in 3T3-L1 cells. The phosphorylation of AMPK level in KBH-1-treated cells was significantly increased compared to that of cells treated with an extract from a single herb alone (S1 Fig). Moreover, in Fig 5, KBH-1-induced the inhibition effect on lipid accumulation and AMPK-mediated signal activation were decreased by blocking AMPK phosphorylation using AMPK siRNA. These results indicate that AMPK phosphorylation is a major pathway for anti-adipogenic effect of KBH-1. The results suggest that the inhibition of adipocyte differentiation by KBH-1 is associated with the regulation of AMPK phosphorylation and that the herb formulation may be able to provide synergistic therapeutic efficacy.

### KBH-1 Administration Dramatically Inhibits Lipid Accumulation without Adverse Effects in Obese Mice

**Fig 6:**
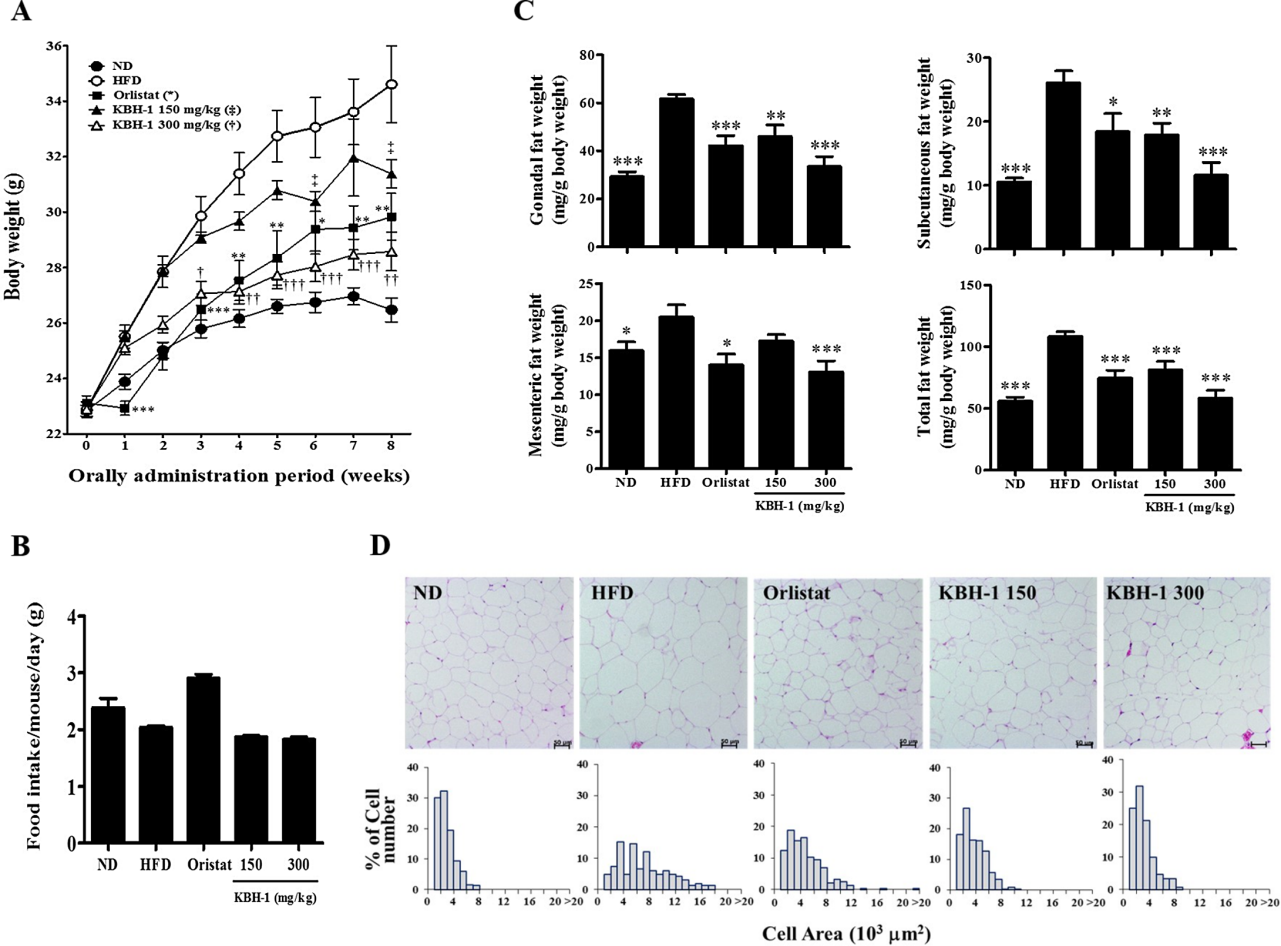
Effects of KBH-1 on high-fat diet (HFD)-induced obese mice. For 8 weeks, **(A)** body weight changes and **(B)** food intake were measured. **(C)** Gonadal fat, subcutaneous fat and mesenteric fat were obtained from mice at the end of the study after an overnight fast and weighed. **(D)** Gonadal adipose tissue was stained with H&E and examined using a light microscope (magnification ×100). The areas of adipocyte distribution from H&E stained gonadal fat sections were expressed in a histogram. Data are expressed as the mean ± SEM (n = 9). Significant differences from the HFD group are indicated by * *p* < 0.05, ** *p* < 0.01 or *** *p* < 0.001.

We confirmed the inhibitory effect of KBH-1 on adipocyte differentiation *in vivo* using a high-fat diet (HFD)-induced obesity mouse model. As shown in Fig 6A, body weight was already higher in mice fed the HFD than in mice fed a normal diet (ND) after the second week, and body weight differences between HFD and ND fed mice gradually increased until the eighth week. In contrast, body weights in the KBH-1 groups were significantly lower than in the HFD group in a dose-dependent manner. In this experiment, orlistat, a well-known anti-obesity drug, was used as a positive control and also reduced the gain in body weight induced by the HFD. During the experimental period, food intake was higher in the ND and orlistat groups than in the HFD group, but did not significantly differ between the HFD and KBH-1 groups (Fig 6B). Furthermore, to identify the anti-obesity effect of KBH-1 on adipose tissue, we measured fat mass and adipocyte size. As expected, the HFD-induced body weight gain was associated with an increase in total fat mass (256%), as well as differential increases in the gonadal fat pad (277%), mesenteric fat pad (172%) and subcutaneous fat pad (325%) indices when compared with the ND control (Fig 6C). However, the KBH-1 groups had significantly lower adipose mass than observed in the HFD group; this difference was dose-dependent. The high-dose (300 mg/kg) treatment with KBH-1 markedly reduced the total fat mass to a value similar to that seen in the ND group (ND 55.8 mg/g body weight, KBH-1 300 58.17 mg/g body weight). As shown in Fig 6D, adipocyte size in HFD-fed mice was markedly enlarged compared to that in ND-fed mice, whereas adipocyte size in the KBH-1 groups was clearly smaller than in the HFD group. Next, we examined blood parameters and organ weight in KBH-1-treated obese mice. To evaluate the effect of KBH-1 on hepatic and renal functions, we determined the plasma parameters (GOT, GPT, ALP, LDH, urea and creatinine) in KBH-1-treated obese mice (S1 Table). However, there were no significant differences in hepatic and renal parameters between the groups. Similarly, neither the organ weights nor the liver histology were significantly different between groups (S2 Table and S2 Fig). Based on these results, KBH-1 treatment showed no liver or kidney toxicity.

### KBH-1 Regulates the AMPK Pathway in HFD-Induced Obese Mice

**Fig 7:**
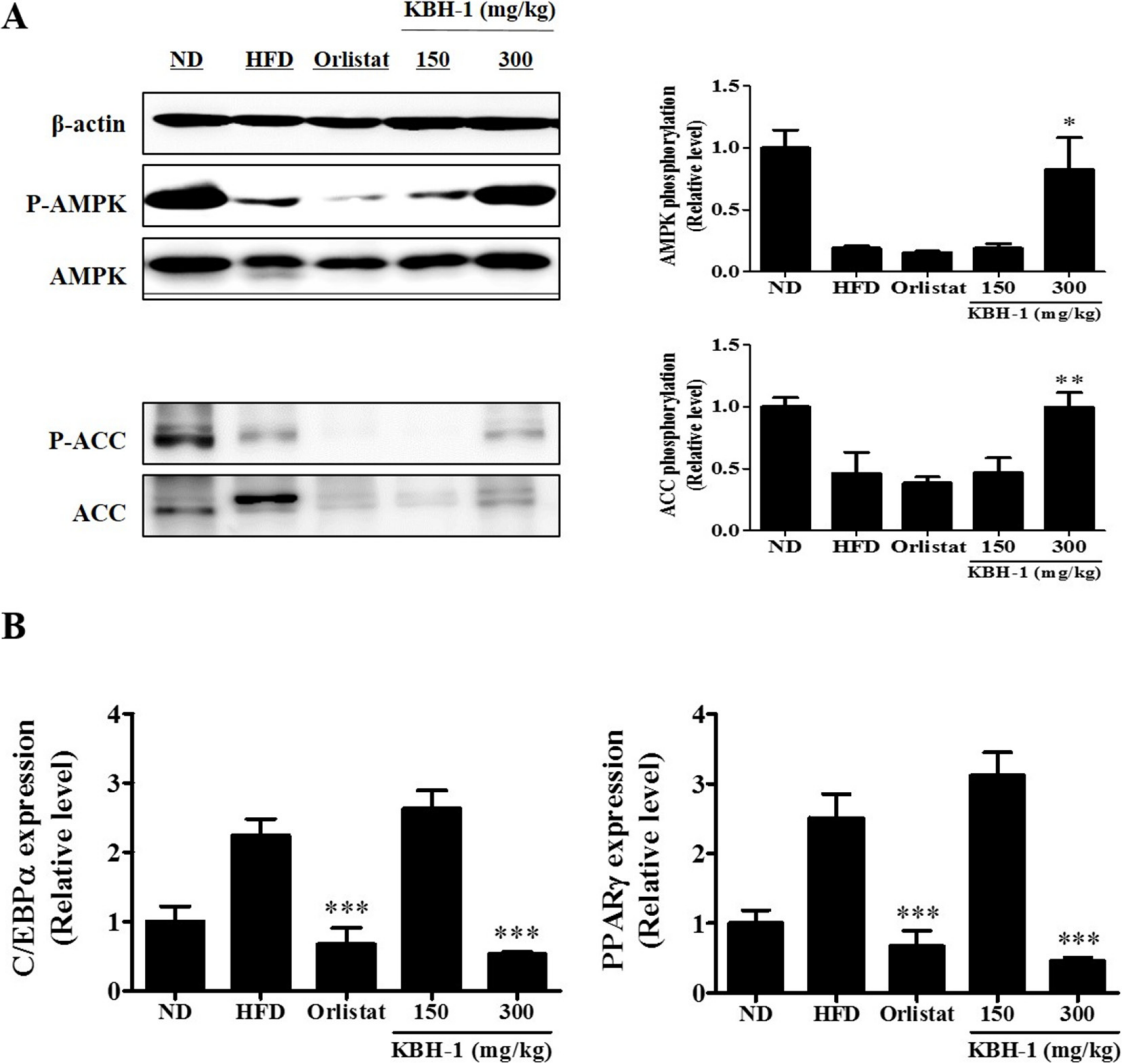
Effects of KBH-1 on AMPK and ACC phosphorylation, and C/EBP α and PPAR γ expression in adipose tissue. **(A)** Gonadal adipose tissue was homogenized, and then the lysates were subjected to western blotting for AMPK and ACC phosphorylation. **(B)** C/EBP α and PPAR γ expression in gonadal adipose tissue were subjected to real-time PCR Data are expressed as the mean ± SEM. Significant differences from HFD group are indicated by * *p* < 0.05, ** *p* < 0.01 or *** *p* < 0.001.

Phosphorylation of AMPK and ACC inhibits lipogenesis [29]. In this study, the phosphorylations of AMPK and ACC were assessed in gonadal adipose tissue. HFD suppressed the phosphorylations of AMPK and ACC, while KBH-1 300 recovered AMPK phosphorylation (Fig 7A). The mRNA levels of C/EBP α and PPAR γ in gonadal adipose tissue were analyzed using real-time PCR. The mRNA expression levels of in C/EBP α and PPAR γ in HFD mice were elevated compared to ND. High-dose treatment with KBH-1 (300 mg/kg) suppressed HFD-induced C/EBP α and PPAR γ gene expression (Fig 7B).

### Characterization of KBH-1 Using HPLC

**Fig 8:**
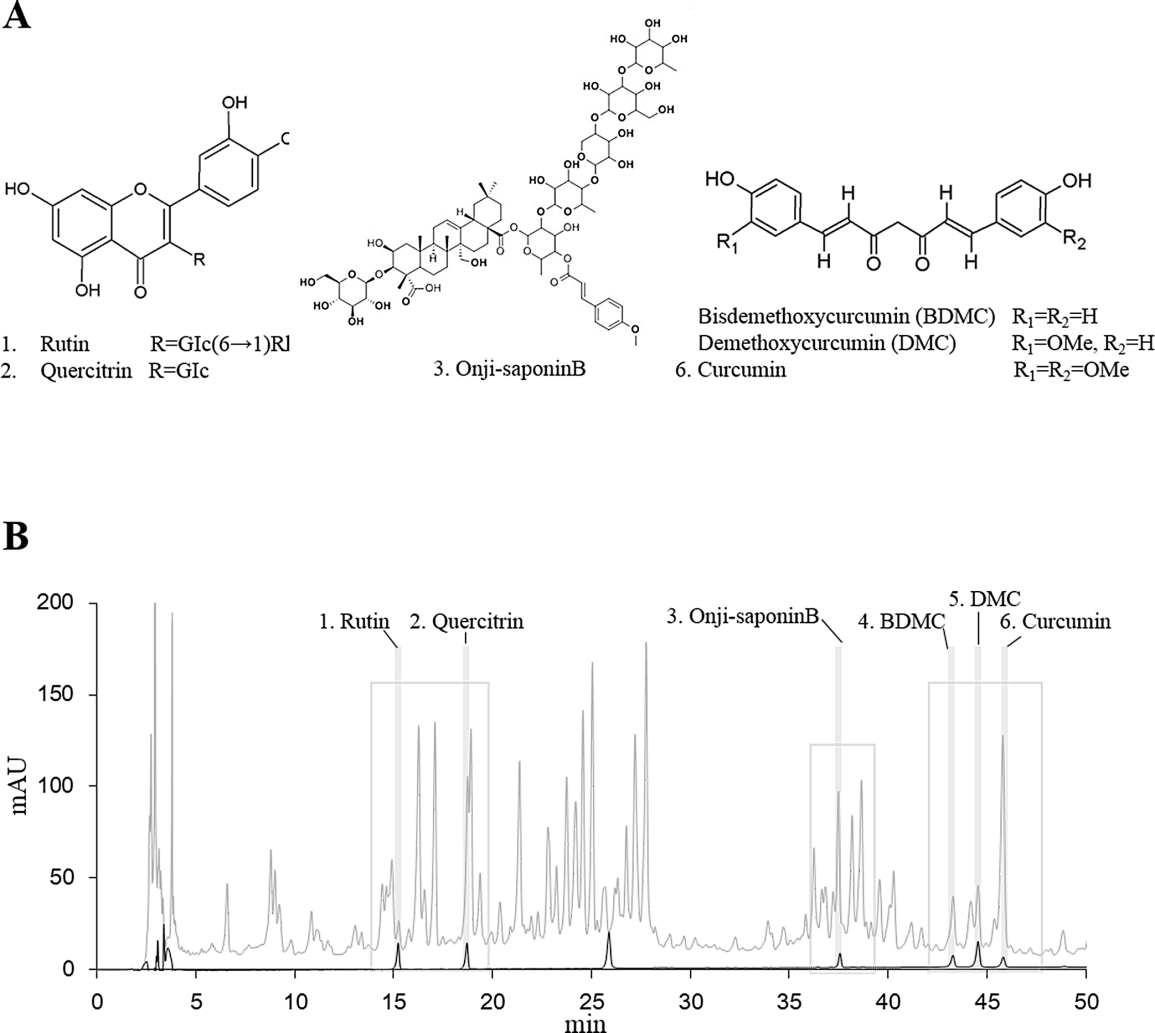
Chemical structures and HPLC profile of the main constituents of KBH-1. The HPLC chromatogram of the components was monitored at 210 nm. Solid and gray lines represent standard compounds and the KBH-1 HPLC profile, respectively.

KBH-1 was analyzed by HPLC using six standard compounds, including rutin, quercitrin, onji-saponin B, BDMC, DMC and curcumin (Fig 8).

## Discussion

Oriental herbal medicines and herbal supplements have been used for weight control. Herbal supplements are cost-effective and exert fewer or no toxic side effects in comparison with many chemically synthesized drugs [30]. In this study, we demonstrated the anti-obesity activity of the herbal medicine KBH-1 in HFD-induced obese mice. These included dose-dependent reductions in body weight, fat mass and size, plasma leptin levels and the expression of adipocyte-specific markers. In addition, KBH-1 treatment inhibited the differentiation of 3T3-L1 preadipocytes through regulation of AMPK pathway. These results provide the first evidence indicating that the new herbal medicine KBH-1 significantly inhibits 3T3-L1 cell differentiation and improves obesity in high fat diet-induced obese mice.

Several studies have shown that adipocyte differentiation and the amount of fat accumulation are associated with the occurrence and development of obesity. Therefore, inhibition of adipocyte differentiation is a strategy in the treatment of obesity. Adipogenesis is a complex process coordinated by the expression of numerous genes. This process is regulated by numerous transcription factors (C/EBPs and PPARs) that activate adipocyte-specific genes. The mouse preadipocyte cell line 3T3-L1 is widely used as an *in vitro* model system to investigate the molecular mechanism of adipogenesis [31]. These cells differentiate into adipocytes and accumulate lipids under adequate *in vitro* culture conditions. We found that the accumulation of TG droplets was significantly decreased by KBH-1 treatment in a dose-dependent manner and without cytotoxicity in 3T3-L1 cells. In the early stages of adipogenic commitment, key transcription factors, such as C/EBP β, are up-regulated. These changes increase the transcription of PPAR γ and C/EBP α [32–34]. We found that KBH-1 reduced the mRNA levels of C/EBP β during adipogenesis and that it significantly inhibited the expression of C/EBP α and PPAR γ. These results suggest that the KBH-1-induced reduction of C/EBP α and PPAR γ expression is dependent on C/EBP β gene expression during adipogenesis. PPAR γ and C/EBP α are major regulators of adipogenesis, activating the transcription of terminal adipocyte differentiation marker genes, such as fabp4, FAS, leptin, adiponectin and IR, and many genes important for triglyceride uptake and storage, such as fabp4, CD36 and LPL [35]. In our study, KBH-1 was able to repress the expression of adipocyte-specific genes, such as leptin, SCD-1, Fabp4 and LPL, and adipocyte-specific proteins, such as LPL, leptin, FAS and Fabp4. These results suggest that KBH-1 inhibits the expression of major transcription factors during adipogenesis, affecting adipocyte differentiation.

Obesity occurs when total energy intake exceeds total energy expenditure, which leads to excessive adipocyte size and/or number. The activation of AMPK, a central sensor of cellular energy, has emerged as a therapeutic target for obesity of which is essential for the inhibition of adipocyte lipogenesis [36, 37]. The activation of AMPK also inhibit adipocyte differentiation and the expression of lipid biosynthetic enzymes such as acetyl-CoA carboxylase (ACC) and fatty acid synthase, the adipogenesis transcription factors PPAR γ and C/EBP α, insulin stimulated glucose transport in adipocyte [38]. In addition, several studies using AMPKα siRNA showed that the AMPK activation affect regulation of PPAR γ, C/EBP α, ACC and SREBP1c expression and adipocyte differentiation [39]. To determine whether KBH-1 inhibits adipocyte differentiation by AMPK phosphorylation, the levels of AMPK, AKT, and ERK1/2 phosphorylation and the cell cycle fractions were determined. The insulin signaling pathway plays an essential role in 3T3-L1 cell differentiation. In the insulin signaling pathway, phosphorylation of ERK and AKT has an important role in differentiation and proliferation [25, 40–42]. However, the results show that AKT and ERK1/2 phosphorylation is not affected by KBH-1 treatment. Only AMPK phosphorylation was increased during the early stage of 3T3-L1 cell differentiation by KBH-1 treatment. AMPK phosphorylation in adipocytes and adipose tissue suppresses lipogenesis through modulation of lipogenic enzymes such as ACC. ACC synthesizes malonyl-CoA from acetyl-CoA and is a key enzyme of the lipogenic pathway [5]. As shown in Fig 4, KBH-1 induced AMPK and ACC phosphorylation in 3T3-L1 cells. Hence, to identify the effect of the activation of AMPK as main target on inhibition of lipid accumulation induced by KBH-1, we investigated using siRNA of AMPK, its results show that the activation of AMPK and the inhibition effect of lipid accumulation were decreased significantly.

*In vivo*, we used a high fat diet-induced obesity mouse model. Obesity is defined as excess adipose mass and adipose tissue expansion. Several studies reported that HFD feeding can increase fat accumulation in adipose tissue. In this study, HFD-induced body weight and fat accumulation in adipose tissue were markedly increased. However, body weight and fat accumulation were markedly reduced by administration of KBH-1. The balance between energy intake and energy expenditure determines energy stores, but some metabolic parameters been found to be predictive of weight gain, such as metabolic rate, spontaneous activity, sympathetic nervous system activity, fat oxidation [43]. Hence, we are ongoing research on the other factors that affect the weight lose by KBH-1 treatment, such as hepatic steatosis in liver and leptin resistance in hypothalamus. The AMPK pathway is involved in the regulation of body weight. Once AMPK is activated, lipogenesis is inhibited, which consequently inhibits fat accumulation [44, 45]. In this study, KBH-1 activated AMPK phosphorylation and inhibited the activities of lipogenic enzyme ACC in gonadal adipose tissue. Several studies have demonstrated that AMPK may be the mediator of hormonal and nutritional effects on adipose tissue by specific translational down-regulation of central transcription factors such as C/EBP α and PPAR γ [26, 46]. In this study, KBH-1 treatment suppressed C/EBP α and PPAR γ gene expression in gonadal adipose tissue. However, it did not affect food intake and ghrelin, a hormone that has a major influence on the regulation of appetite. Furthermore, the administration of KBH-1 for 8 weeks did not cause death or abnormal behavior. Organ weights, hepatic histology and the levels of AST, ALT, ALP and BUN, CRE were not significantly different in mice administered KBH-1 compared with those of the control group, suggesting that KBH-1 administration did not cause hepatic or renal damage. These results sugguest the KBH-1 did not affect the toxicity in the liver and kidney. In addition, to identify an active component of anti-obesity of KBH-1, six chemical compounds, such as rutin, quercitrin, onji-saponin B, BDMC, DMC and curcumin, were analyzed by HPLC. Presently, as further study, we have investigating follow-up study including the anti-obesity effect of active compounds and its possible mechanism.

The results of this study suggest that the new herbal drug KBH-1 inhibits lipid accumulation and adipogenesis in 3T3-L1 cells and reduces body weight and fat accumulation in HFD-induced obese mice. Furthermore, our results demonstrated that KBH-1 inhibits lipid accumulation by regulating the transcriptional factors and their downstream lipogenic targets via the activation of AMPK pathway. 3T3-L1 cell viability, as well as liver, kidney, spleen and testis weights and serum chemistry levels in experimental mice were not affected by KBH-1 treatment. These results indicate that KBH-1 could be developed as a new naturally occurring therapeutic anti-obesity herbal medicine without any toxic effects.

## Supporting Information

S1 Fig Effects of KBH-1, *Polygala tenuifolia* (PT), *Saururus chinensis* (SC) and *Curcuma* longa (CL) on AMPK activation Differentiated preadipocytes treated with 30 ug/ml of KBH-1, 10 ug/ml of KBH-1, PT, SC and CL for 120 min were subjected to Western blotting to determine the levels of AMPK phosphoryalted form. The band intensities relative to those of the untreated "0 min" cells were determined after normalizing to total form expression and represented as the mean ± SEM. Significant differences from (DM) are indicated by **p < 0.01 or ***p < 0.001.
(TIF)

S2 Fig Effect of KBH-1 on liver tissue. Liver tissue was obtained from mice after fasting overnight at the end of the study, and stained with H&E and examined using a light microscope (magnification ×100).
(TIF)

S1 Table Effect of KBH-1 on serum chemical analysis. (DOCX)

S2 Table Effect of KBH-1 on organ weights. (DOCX)

S1 Text Supplementary data. (DOCX)
