# Supplementary figures and images for: The Herbal Medicine KBH-1 Inhibits Fat Accumulation in 3T3-L1 Adipocytes and Reduces High Fat Diet-Induced Obesity through Regulation of the AMPK Pathway
Source: PLoS One. 2015 Dec 9;10(12):e0142041. doi: 10.1371/journal.pone.0142041 (PMC4674115; doi:10.1371/journal.pone.0142041)

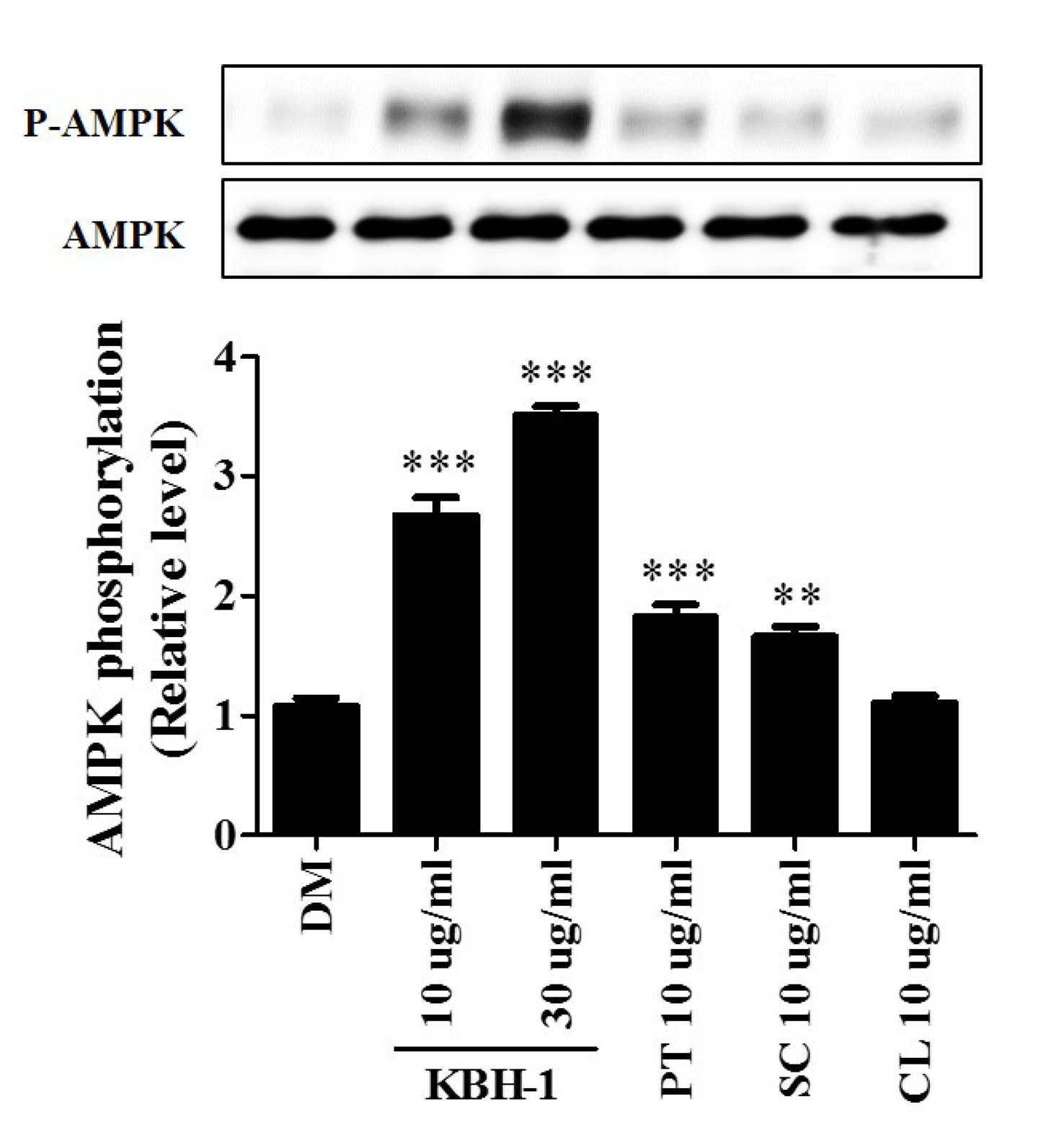

Supplement: S1 Fig — The band intensities relative to those of the untreated "0 min" cells were determined after normalizing to total form expression and represented as the mean ± SEM. Significant differences from (DM) are indicated by **p < 0.01 or ***p < 0.001. (TIF) [file pone.0142041.s001.tif]

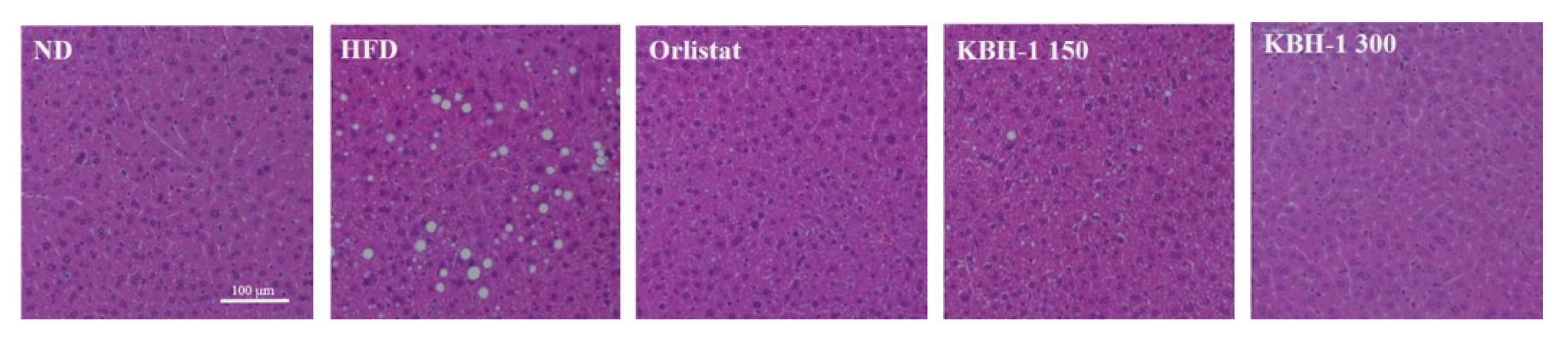

Supplement: S2 Fig — Liver tissue was obtained from mice after fasting overnight at the end of the study, and stained with H&E and examined using a light microscope (magnification ×100). (TIF) [file pone.0142041.s002.tif]
