# Supplementary material for: The Herbal Medicine KBH-1 Inhibits Fat Accumulation in 3T3-L1 Adipocytes and Reduces High Fat Diet-Induced Obesity through Regulation of the AMPK Pathway
Source: PLoS One. 2015 Dec 9;10(12):e0142041. doi: 10.1371/journal.pone.0142041 (PMC4674115; doi:10.1371/journal.pone.0142041)
Supplement: S1 Table — (DOCX) [file pone.0142041.s003.docx]

**S1 Table. Effect of KBH-1 on serum chemical analysis.**

|  | **ND** | **HFD** | **Orlistat** | **KBH-1 150** | **KBH-1 300** |
| --- | --- | --- | --- | --- | --- |
| Leptin (ng/ml) | 1.94±1.85 | 12.14±1.74 | 4.32±0.79*** | 6.85±1.53** | 2.82±1.01*** |
| Hepatic function (IU/l) : | | | | | |
| GOT | 103.33±10.67 | 86.67±10.90 | 108.33±10.40 | 97.14±15.46 | 98.33±16.16 |
| GPT | 25.63±3.46 | 26.11±3.31 | 26.11±1.62 | 24.29±2.97 | 25.56±2.56 |
| ALP | 76.11±12.98 | 65.00±9.20 | 62.22±5.47 | 56.43±10.79 | 62.78±7.08 |
| Renal function (mg/dl) : | | | | | |
| Urea | 23.89±1.70 | 19.89±1.12 | 18.83±1.01 | 23.21±1.79 | 22.94±1.60 |
| Creatinine | 4.00±0.61 | 3.72±0.55 | 3.67±0.55 | 4.00±0.66 | 3.94±0.67 |

Values are expressed as the mean ± SEM (n=9). Significant differences from HFD group are indicated by ***p* < 0.01 or ****p* < 0.001.
