# Supplementary material for: The Herbal Medicine KBH-1 Inhibits Fat Accumulation in 3T3-L1 Adipocytes and Reduces High Fat Diet-Induced Obesity through Regulation of the AMPK Pathway
Source: PLoS One. 2015 Dec 9;10(12):e0142041. doi: 10.1371/journal.pone.0142041 (PMC4674115; doi:10.1371/journal.pone.0142041)
Supplement: S2 Table — (DOCX) [file pone.0142041.s004.docx]

**S2 Table. Effect of KBH-1 on organ weights.**

| **Organ (g)** | | **ND** | **HFD** | **Orlistat** | **KBH-1 150** | **KBH-1 300** |
| --- | --- | --- | --- | --- | --- | --- |
| Liver | | 0.920±0.031 | 0.981±0.045 | 0.979±0.034 | 1.045±0.035 | 1.028±0.017 |
| Heart | | 0.122±0.005 | 0.125±0.007 | 0.122±0.004 | 0.133±0.005 | 0.133±0.005 |
| Lung | | 0.152±0.008 | 0.170±0.006 | 0.159±0.004 | 0.167±0.008 | 0.149±0.006 |
| Spleen | | 0.071±0.005 | 0.081±0.007 | 0.074±0.004 | 0.077±0.004 | 0.079±0.011 |
| Kidney | R | 0.136±0.005 | 0.156±0.005 | 0.153±0.007 | 0.161±0.006 | 0.147±0.004 |
|  | L | 0.135±0.002 | 0.152±0.008 | 0.151±0.006 | 0.154±0.005 | 0.142±0.006 |
| Testis | R | 0.096±0.006 | 0.101±0.005 | 0.096±0.004 | 0.106±0.004 | 0.094±0.004 |
|  | L | 0.097±0.004 | 0.097±0.004 | 0.096±0.005 | 0.100±0.004 | 0.091±0.005 |

Values are expressed as the mean ± SEM (n=9).
