# Supplementary material for: The Herbal Medicine KBH-1 Inhibits Fat Accumulation in 3T3-L1 Adipocytes and Reduces High Fat Diet-Induced Obesity through Regulation of the AMPK Pathway
Source: PLoS One. 2015 Dec 9;10(12):e0142041. doi: 10.1371/journal.pone.0142041 (PMC4674115; doi:10.1371/journal.pone.0142041)
Supplement: S1 Text — (DOCX) [file pone.0142041.s005.docx]

**Supplementary data**

**Method**

**Protein extraction and Western blotting**

Samples were lysed in Ripa buffer consisting of 50 mM Tris-HCl (pH 8.0), 5 mM EDTA, 150 mM NaCl, 1% NP-40, 0.1% SDS, 1 mM PMSF, one protease-inhibitor cocktail tablet and one phosphatase-inhibitor cocktail tablet. Cell lysates were centrifuged at 13,000 rpm for 30 min at 4℃. Protein concentration was determined with a BCA Protein Assay Kit. Protein samples were mixed with sample buffer (100 mM Tris-HCl [pH 7.6], 2% SDS, 1% 2-mercaptoethanol, 2% glycerol and 0.01% bromophenol blue) and incubated at 97℃ for 5 min. Aliquots of 15 μg of protein extract were loaded onto 8~15 % polyacrylamide gels. Electrophoresis was performed using the Mini Protein 3 Cell (Bio-Rad, Hercules, CA, USA). Resolved proteins were transferred onto a PVDF membrane. The membrane was first incubated in blocking buffer (10 mM Tris-HCl [pH 7.5], 150 mM NaCl, 0.1% Tween 20 and 3% BSA) and then incubated overnight at 4℃ with 1000 diluted primary antibodies. After washing with washing buffer (10 mM Tris-HCl [pH 7.5], 150 mM NaCl and 0.1% Tween 20) three times for 20 min each, the membrane was probed with 5000 diluted secondary antibodies for 1 h at room temperature. The membrane was then washed with washing buffer three times for 10 min each and developed with an ECL kit. Chemiluminescent signals were detected using a LAS-4000 Luminescent Image Analyzer (Fuji Photo Film Co., Japan). Band intensities were quantified using ImageJ software (National Institutes of Health, USA).

**High-fat diet (HFD)-induced obesity mouse model**

All animal experiments were conducted in accordance with the Guide for the Care and Use of Laboratory Animals of the National Institute of Health (NIH publication No. 83-23, revised 1996) and were approved by the Institutional Animal Care and Use Committee of the KIOM. Male C57BL/6N mice were purchased from Samtako. All mice were housed in a room with controlled temperature (20­–24℃), humidity (40­–60%) and lighting (12 h light/dark cycle) and were supplied with water *ad libitum*. After acclimation for 1 week, mice were randomly divided into five groups of nine mice each: (1) ND: 10% kcal fat diet fed mice; (2) HFD: 60% kcal fat diet fed mice; (3) orlistat: HFD treated with 75 mg/kg body weight/day orlistat administration; (4) KBH-1 150: HFD treated with 150 mg/kg body weight/day KBH-1 extract administration; and (5) KBH-1 300: HFD treated with 300 mg/kg body weight/day KBH-1 extract administration. 10% kcal fat or 60% kcal fat diets were provided to mice for 8 weeks. Oral administration of each sample occurred once a day during the diet period. The same amount of saline was orally administered to the control groups. At the end of the experimental period, mice were euthanized, blood was collected and organs were excised. The organs were rinsed with saline solution, weighed and stored at -80℃.

**Measurement of blood biochemical parameters**

All mice were euthanized using avertin after overnight fasting. Blood was collected from the abdominal vena cava and divided into 2 parts: serum and plasma. Serum was taken to determine glutamic-oxaloacetic transaminase (GOT), glutamic pyruvic transaminase (GPT), lactate dehydrogenase (LDH), urea, creatinine and alkaline phosphatase (ALP) levels using an automatic analyzer. Plasma was used to determine leptin and ghrelin levels using a commercial ELISA kit according the manufacturer’s protocol.

**Histological analysis**

Gonadal adipose tissue was dissected and immediately fixed in 4% neutral formaldehyde solution. Tissues were subsequently dehydrated in a graded ethanol series (70­–100%) and embedded in paraffin. The tissues were sectioned (4 μm thick) with a Leica RM 2165 rotary microtome (Wetzlar, Germany) and stained with hematoxylin and eosin (H&E). Sections were viewed with an Axioskop 40 (Oberkochen, Germany) and photographed at 100 X magnification.

**Results**

**KBH-1 regulates the AMPK pathway in 3T3-L1**

To identify the synergic effect of KBH-1 through AMPK activation, KBH-1 and composition drug, *Polygala tenuifolia* (PT), *Saururus chinensis* (SC) and *Curcuma longa* (CL), were treated with adipocyte differentiation medium in 3T3-L1 cells. The phosphorylation of AMPK level of KBH-1 were significantly increased compared to composition drug (S1 Fig.). The results suggested that the inhibition of adipocyte differentiation by KBH-1 was associated with the regulation of AMPK phosphorylation and the herb formulation may be able to deliver synergistic therapeutic efficacy.

**KBH-1 administration inhibits plasma leptin in obesity mice without adverse effects**

As shown in S1 Table, HFD-induced plasma leptin levels were elevated by approximately 8.8-fold compared to the ND group. The leptin levels of the Orlistat, KBH-1 150 and KBH-1 300 groups were significantly decreased by approximately 84.01%, 57.87% and 80.34%, respectively, compared to the HFD group. To evaluate the effect of KBH-1 on hepatic and renal functions, we determined the plasma parameters (GOT, GPT, ALP, LDH, urea and creatinine) in KBH-1-treated obese mice. However, none hepatic and renal parameters differed significantly among the groups.

**KBH-1 did not affect the toxicity in the liver and kidney.**

We confirmed the toxicity activity of KBH-1 on adipocyte differentiation *in vivo* using a high-fat diet (HFD) induced obesity mouse model. As shown in S2 Table and S2 Fig., none of the organ weights and liver histology were significantly different between groups. Based on these results, KBH-1 treatment showed no liver or kidney toxicity.

**S1 Table. Effect of KBH-1 on serum chemical analysis.**

|  | **ND** | **HFD** | **Orlistat** | **KBH-1 150** | **KBH-1 300** |
| --- | --- | --- | --- | --- | --- |
| Leptin (ng/ml) | 1.94±1.85 | 12.14±1.74 | 4.32±0.79*** | 6.85±1.53** | 2.82±1.01*** |
| Hepatic function (IU/l) : | | | | | |
| GOT | 103.33±10.67 | 86.67±10.90 | 108.33±10.40 | 97.14±15.46 | 98.33±16.16 |
| GPT | 25.63±3.46 | 26.11±3.31 | 26.11±1.62 | 24.29±2.97 | 25.56±2.56 |
| ALP | 76.11±12.98 | 65.00±9.20 | 62.22±5.47 | 56.43±10.79 | 62.78±7.08 |
| Renal function (mg/dl) : | | | | | |
| Urea | 23.89±1.70 | 19.89±1.12 | 18.83±1.01 | 23.21±1.79 | 22.94±1.60 |
| Creatinine | 4.00±0.61 | 3.72±0.55 | 3.67±0.55 | 4.00±0.66 | 3.94±0.67 |

Values are expressed as the mean ± SEM (n=9). Significant differences from HFD group are indicated by ***p* < 0.01 or ****p* < 0.001.

**S2 Table. Effect of KBH-1 on organ weights.**

| **Organ (g)** | | **ND** | **HFD** | **Orlistat** | **KBH-1 150** | **KBH-1 300** |
| --- | --- | --- | --- | --- | --- | --- |
| Liver | | 0.920±0.031 | 0.981±0.045 | 0.979±0.034 | 1.045±0.035 | 1.028±0.017 |
| Heart | | 0.122±0.005 | 0.125±0.007 | 0.122±0.004 | 0.133±0.005 | 0.133±0.005 |
| Lung | | 0.152±0.008 | 0.170±0.006 | 0.159±0.004 | 0.167±0.008 | 0.149±0.006 |
| Spleen | | 0.071±0.005 | 0.081±0.007 | 0.074±0.004 | 0.077±0.004 | 0.079±0.011 |
| Kidney | R | 0.136±0.005 | 0.156±0.005 | 0.153±0.007 | 0.161±0.006 | 0.147±0.004 |
|  | L | 0.135±0.002 | 0.152±0.008 | 0.151±0.006 | 0.154±0.005 | 0.142±0.006 |
| Testis | R | 0.096±0.006 | 0.101±0.005 | 0.096±0.004 | 0.106±0.004 | 0.094±0.004 |
|  | L | 0.097±0.004 | 0.097±0.004 | 0.096±0.005 | 0.100±0.004 | 0.091±0.005 |

Values are expressed as the mean ± SEM (n=9).

**Figure legends**

**S1 Fig. Effects of KBH-1, *Polygala tenuifolia* (PT), *Saururus chinensis* (SC) and *Curcuma* longa (CL) on AMPK activation.** Differentiated preadipocytes treated with 30 ug/ml of KBH-1, 10 ug/ml of KBH-1, PT, SC and CL for 120 min were subjected to Western blotting to determine the levels of AMPK phosphorylated form. The band intensities relative to those of the untreated "0 min" cells were determined after normalizing to total form expression and represented as the mean ± SEM. Significant differences from (DM) are indicated by ***p* < 0.01 or ****p* < 0.001.

**S2 Fig. Effect of KBH-1 on liver tissue.** Liver tissue was obtained from mice after fasting overnight at the end of the study, and stained with H&E and examined using a light microscope (magnification ×100).
